# Transversus Abdominis Plane Block Versus Rectus Sheath Block for Postoperative Pain After Caesarean Delivery: A Randomised Controlled Trial

**DOI:** 10.5152/TJAR.2023.22724

**Published:** 2023-02-01

**Authors:** Hadi Ufuk Yörükoğlu, Tülay Şahin, Ayşe Öge Kula

**Affiliations:** 1 Department of Anaesthesiology and Reanimation, Kocaeli University Faculty of Medicine, Kocaeli, Turkey; 2 Department of Anaesthesiology, Medical College of Wisconsin, Milwaukee, Wisconsin, USA

**Keywords:** Caesarean delivery, obstetric anaesthesia, postoperative analgesia, RS block, TAP block

## Abstract

**Objective:**

Postoperative analgesia in caesarean deliveries is becoming increasingly important, since early bonding between mother and infant can be established with effective postoperative analgesia while preventing the unpleasant effects of pain. Additionally, inadequate postoperative analgesia is associated with chronic pain and postpartum depression. The primary objective of this study was to compare the analgesic effects of transversus abdominis plane block and rectus sheath block in patients undergoing elective caesarean delivery.

**Methods:**

A total of 90 parturients with American Society of Anesthesia status I-II, aged 18-45 years, at >37 gestational weeks, and scheduled for elective caesarean delivery were included in the study. All patients received spinal anaesthesia. Parturients were randomised into 3 groups. Bilateral ultrasound-guided transversus abdominis plane block was performed on the transversus abdominis plane group, bilateral ultrasound-guided rectus sheath block on the rectus sheath group, and no block on the control group. All patients were given intravenous morphine through a patient-controlled analgesia device. A pain nurse, blinded to the study, recorded the cumulative morphine consumption and pain scores during resting and coughing using a numerical rating scale at postoperative hours 1, 6, 12, and 24.

**Results:**

Numerical rating scale values recorded during rest and coughing were lower in the transversus abdominis plane group at postoperative hours 2, 3, 6, 12, and 24 (*P* < .05). Morphine consumption was lower in the transversus abdominis plane group at postoperative hours 1, 2, 3, 6, 12, and 24 (*P* < .05).

**Conclusion:**

Transversus abdominis plane block provides effective postoperative analgesia in parturients. However, rectus sheath block provides inadequate postoperative analgesia in parturients who undergo caesarean delivery.

**Main Points**

- Transversus abdominis plane block provides effective analgesia in parturients who undergo elective caesarean delivery.
- The postoperative analgesic effect of rectus sheath block is inadequate in parturients who undergo elective caesarean delivery.
- Regional anaesthesia techniques should be performed as a multimodal analgesia regimen to provide effective analgesia in caesarean delivery.

## Introduction

Caesarean delivery is one of the most frequently performed surgeries in the world. Postoperative analgesia in caesarean delivery is becoming increasingly important because early bonding between mother and infant can be established with effective postoperative analgesia while preventing the unpleasant effects of pain. Additionally, inadequate postoperative analgesia is associated with chronic pain and postpartum depression.^1^

Numerous methods are used for the treatment of postoperative pain. Opioids can be administered via the intravenous route, in a neuraxial manner, or both. However, the side effects of opioids include nausea, vomiting, urinary retention, and respiratory depression. Regional anaesthesia techniques are becoming increasingly frequently used in order to reduce opioid consumption and provide more effective postoperative analgesia.

With the use of ultrasound, numerous regional analgesia techniques have been described and performed with minimal complication risks. Transversus abdominis plane (TAP) block is an effective technique for caesarean delivery.^2^ Rectus sheath (RS) block is commonly performed for lower and upper abdominal surgeries and provides effective analgesia. However, to the best of our knowledge, there have been no randomised controlled trials for its use in caesarean delivery.^3^

The primary goal of this study was to compare the postoperative analgesic effects of TAP and RS blocks and opioid consumption levels. Secondary goals were to compare nausea, vomiting, and patient satisfaction levels.

## Methods

This prospective, randomised controlled study was performed following the receipt of Kocaeli City Clinical Trials Ethical Committee, Turkey, approval (KIA 2017/348) and written informed consent from the patients. The study was conducted between March and August 2018.

Parturients aged 18-45 years with American Society of Anesthesia (ASA) physical status I-II were included in the study. Exclusion criteria were obesity (body mass index >35 kg m^2–1^), presence of foetal distress, gestational age <37 weeks, skin infection at the needle puncture site, known allergy to any of the study drugs, coagulopathy, recent use of any analgesic drugs or magnesium, or inability to comprehend or use the numerical rating pain scoring system or patient-controlled analgesia (PCA) pump.

Randomisation was achieved using the sequentially numbered opaque sealed envelope technique. Patients were randomized into 1 of 3 groups—TAP, RS, and control. Transversus abdominis plane block was performed postoperatively on the TAP group patients and RS block postoperatively on the RS group. Control group patients received no intervention. All blocks were performed by experienced anaesthesiologists (T.Ş. and H.U.Y.) blinded to the data collection.

In the operating room, all patients underwent standardised monitoring including pulse oximeter (SpO_2_), electrocardiography (ECG), and noninvasive blood pressure monitoring. NaCl 0.9% infusion was set to 10 mL^–1^ kg^–1^ h^–1^, and 6 L min^–1^ oxygen was provided. Patients received spinal anaesthesia with 8 mg hyperbaric bupivacaine and 25 µg fentanyl in the lateral decubitus position at the L4-L5 interspace. Surgery commenced once an upper sensory level of T6 or higher, tested with a pinprick, had been achieved. If the upper sensory level was below T6 after 20 minutes, this was regarded as failed spinal anaesthesia, and the patient would be excluded from the study.

At the end of the surgery, paracetamol 1 g was administered intravenously to all patients. In the recovery room, all patients were given a PCA device with morphine 0.5 mg mL^–1^, set to deliver a 1 mg bolus dose, with an 80 minute lockout time and a 6 mg 1 h limit. Paracetamol 1 mg was also routinely administered every 6 hours for every patient on the ward. Rescue analgesia with tenoxicam 20 mg intravenously was administered in case of numerical rating scale (NRS) values >3.

In the TAP group, after completion of surgery, bilateral ultrasound-guided TAP block was performed using a linear probe (Esaote MyLab 5i, Florence, Italy). The probe was placed transversely between the iliac crest and costal margin in the anterior axillary line and slid in a medial–lateral direction in order to visualise the external oblique, internal oblique, and transversus abdominis muscles. The block was performed in the mid-axillary line. The needle was inserted with a medial to lateral in-plane approach, and 20 mL of bupivacaine 0.25% was injected under direct visualisation in the plane between the transversus abdominis muscle and the fascia deep to the internal oblique muscle on each side.

In the RS group, after completion of surgery, bilateral ultrasound-guided RS block was performed using a linear probe. This was placed transversely at the lateral side of the umbilicus and slid in a lateral direction in order to visualise the rectus muscle, RS, and external and internal oblique muscles. The injection area was defined as the site where the optimal visualisation of the posterior RS was obtained. The needle was inserted with an in-plane approach, and 20 mL of bupivacaine 0.25% was injected bilaterally into the RS.

The patients’ gestational weeks, duration of surgery, and upper dermatome of spinal anaesthesia were recorded. A pain nurse, blinded to the study, recorded cumulative morphine consumption at postoperative hours 1, 6, 12, and 24. Postoperative pain was assessed using an NRS ranging from 0 (no pain) to 10 (worst imaginable pain) at postoperative hours 1, 6, 12, and 24 at rest and while coughing. The pain nurse also recorded the incidence of nausea and vomiting in the postoperative first 24 hours and investigated the patients’ satisfaction, ranging from 0 (unsatisfied) to 4 (very satisfied).

### Statistical Analysis

A preliminary study in our clinic involving 10 patients showed that for 80% power and an error of 0.05, the sample size necessary to detect a 30% difference in morphine consumption at the 24th hour would be 28 subjects for each group. We included 32 patients in each group against the possibility of dropouts.

All statistical analyses were performed on IBM Statistical Package for the Social Sciences for Windows version 20.0 (IBM Corp., Armonk, NY, USA). The Kolmogorov–Smirnov test was used to assess the normality of the data distribution. Continuous variables were expressed as mean ± SD and median (25th-75th percentiles) values and categorical variables as counts (percentages). Normally distributed continuous variables were compared between the groups and were performed using 1-way analysis of variance (ANOVA) and Tukey’s post hoc test.

Non-normally distributed continuous variables were compared between the groups using the Kruskal–Wallis 1-way ANOVA and the Dunn’s post hoc test. Non-normally distributed continuous variables were compared between the time points using the Friedman ANOVA by ranks and the Tukey’s post hoc test. Two-sided *P* <.05 were considered statistically significant.

## Results

**Figure 1.**
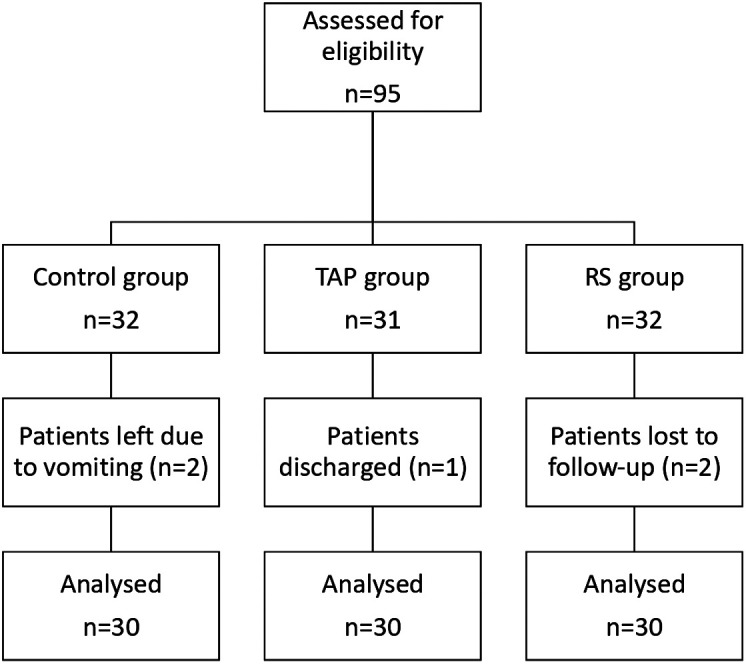
Consort flow diagram. RS, rectus sheath; TAP, transversus abdominis plane.

Ninety patients were recruited for the study (Figure 1). Demographic data, gestational weeks, ASA physical status, duration of surgery, and upper dermatomes of spinal anaesthesia were similar between the groups (Table 1).

**Figure 2.**
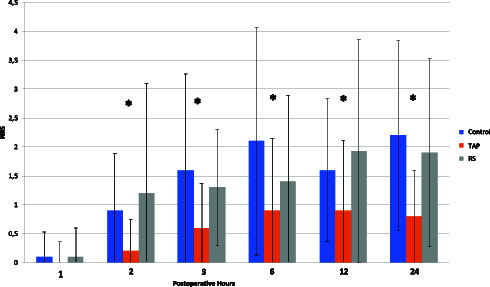
NRS scores while resting at postoperative hours 1, 2, 3, 6, 12, and 24. Comparisons of non-normally distributed continuous variables between the groups were performed using Kruskal–Wallis 1-way ANOVA and the Dunn’s post hoc test (data presented as mean ± SD). Comparisons of non-normally distributed continuous variables between the time points were performed using Friedman ANOVA by ranks and the Tukey’s post hoc test. *TAP group compared with the RS and control groups (*P* < .05). ANOVA, analysis of variance; NRS, numerical rating scale; RS, rectus sheath; TAP, transversus abdominis plane.

**Figure 3.**
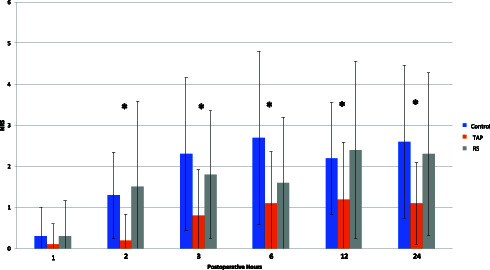
NRS scores while coughing at postoperative hours 1, 2, 3, 6, 12, and 24. Comparisons of non-normally distributed continuous variables between the groups were performed using Kruskal–Wallis 1-way ANOVA and the Dunn’s post hoc test (data presented as mean ± SD). Non-normally distributed continuous variables were compared between the different time points using Friedman ANOVA by ranks and the Tukey’s post hoc test. *TAP group compared with RS and control group (*P* < .05). ANOVA, analysis of variance; NRS, numerical rating scale; RS, rectus sheath; TAP, transversus abdominis plane.

Numerical rating scale scores while resting and coughing at postoperative hour 1 were similar between the groups. However, NRS scores while resting at postoperative hours 2, 3, 6, 12, and 24 were significantly lower in the TAP group compared with the RS and control groups (*P* < .05) (Figure 2). Numerical rating scale scores while coughing at postoperative hours 2, 3, 12, and 24 were significantly lower in the TAP group compared with the RS and control groups (*P* < .05) (Figure 3).

**Figure 4.**
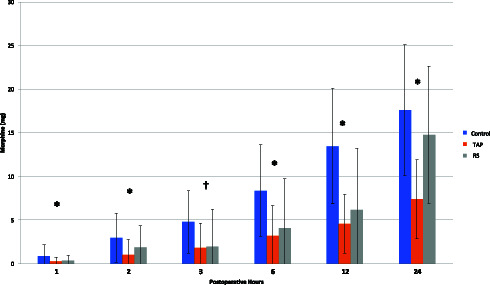
Morphine consumption at postoperative hours 1, 2, 3, 6, 12, and 24. Non-normally distributed continuous variables were compared between the groups using Kruskal–Wallis 1-way ANOVA and the Dunn’s post hoc test (data presented as mean ± SD). Non-normally distributed continuous variables were compared between the different time points using Friedman ANOVA by ranks and the Tukey’s post hoc test. *TAP group compared with the RS and control groups (*P* < .05). †TAP and RS groups compared with the control group (*P* < .05). ANOVA, analysis of variance; RS, rectus sheath; TAP, transversus abdominis plane.

Total morphine consumption was significantly lower in the TAP group compared with the RS and control groups (*P* < .05). Morphine consumption was significantly lower only at postoperative hour 3 in the RS group compared with the control group (*P* < .05) (Figure 4).

Patient satisfaction was higher in both the TAP and RS groups compared with the control group (*P* < .001).

Three patients in the control group, 1 in the TAP group, and 4 in the RS group experienced nausea. One patient in both the RS and control groups experienced vomiting.

No respiratory depression occurred in any patients.

## Discussion

The primary objective of this study was to compare the postoperative analgesic effect of RS block with that of TAP block in parturients undergoing caesarean delivery. At the postoperative first hour, NRS scores and morphine consumptions were similar in all groups due to the effect of spinal anaesthesia. However, NRS scores and morphine consumption were lower in the TAP group compared with the RS and control groups overall.

Numerous studies have evaluated the efficiency of TAP block in caesarean delivery under both general and spinal anaesthesia.^2,4-9^ In general, spinal anaesthesia is preferred for caesarean delivery except in conditions in which spinal anaesthesia is contraindicated.

Many methods have been described for TAP block. Faiz et al^10^ showed that the posterior approach is more effective than the lateral approach in caesarean delivery. We also employed the posterior approach in the present study. A review of 14 studies which 20 mL of local anaesthetic was used described lower doses (<50 mg bupivacaine or equivalent) as effective.^11^ In the present study, we used 50 mg bupivacaine (20 mL) for both TAP and RS blocks for each side.

Baaj et al^2^ reported that bilateral ultrasound-guided TAP block reduces morphine consumption by 60% and increases patient satisfaction compared with a placebo group in caesarean delivery under spinal anaesthesia using intrathecal bupivacaine and fentanyl. Loane et al^7^ compared intrathecal morphine (100 µg) with bilateral TAP block (with 20 mL of 0.5% ropivacaine). Although visual analogue scale scores were lower with intrathecal morphine, the adverse effects of morphine were significantly higher. Similarly, Kanazi et al^9^ reported that intrathecal morphine is more effective than bilateral TAP block. This may be due to the prolonged analgesic effect of intrathecal morphine, although adverse effects such as late respiratory depression should also be kept in mind. Transversus abdominis plane block reduced morphine consumption by 55% in the present study, and the incidences of nausea and vomiting were lower in the TAP group compared with the RS group, although the difference was statistically insignificant. 

Transversus abdominis plane block has also been compared with other techniques. Some studies have found that the wound infiltration technique produces a similar analgesic effect,^12,13^ while Aydogmus et al^14^ and Görkem et al^15^ showed that TAP block results in lower opioid consumption than wound infiltration.

Rectus sheath block targets the anterior cutaneous branches of the thoracoabdominal nerves and terminal muscular branches and provides somatic analgesia for abdominal surgeries in a range of laparoscopic and open procedures, including major gynaecological surgery.^16^

Although many studies have shown the efficacy of RS block for postoperative analgesia in abdominal surgeries, few studies have investigated it in caesarean delivery. In 1 previous study, RS block was performed by the surgeon before closure using bupivacaine or saline, and no difference in postoperative pain scores was observed between the groups.^17^ However, the patients in that study received intrathecal morphine, PCA devices were not used, and opioid consumption was not compared. Cüneyitoğlu et al^18^ performed RS block with 20 mL 0.25% bupivacaine for postoperative analgesia in gynaecological surgeries using Pfannenstiel incisions under general anaesthesia and described the block as effective. However, the analgesic effect of RS block in the present study was not statistically significant.

Different injection points have been described for RS block. One cadaver study suggested that it might be more effective to inject the local anaesthetic between the rectus abdominis fascia and transversalis fascia.^19^ The local anaesthetic was also injected in this area in the present study.

There are several limitations to this study. First, blocks were performed after completion of caesarean delivery. It was not therefore possible to perform sensory testing for mapping of the block area. In addition, no sham block group was established due to ethical concerns, which may also be considered a limitation.

In conclusion, TAP block provides effective analgesia in parturients undergoing elective caesarean delivery. Rectus sheath block reduced opioid consumption, although this was statistically insignificant, and the postoperative analgesia effect of the block is inadequate.

## Figures and Tables

**Table 1.** Comparisons of Normally Distributed Continuous Variables Between the Groups Using 1-Way Analysis of Variance and Tukey’s Post Hoc Test

	Control Group	TAP Group	RS Group	*P*
	(n = 30)	(n = 30)	(n = 30)	
Age (years)	31.5 ± 4.48	32.2 ± 5.58	31.4 ± 5.01	.821
Weight (kg)	76.3 ± 10.23	76.1 ± 7.43	75.9 ± 8.99	.983
Height (cm)	162 ± 5.19	162 ± 5.14	163 ± 5.93	.551
ASA I/II (n)	28/2	23/7	23/7	.178
Duration of surgery (min)	60.6 ± 11.57	57 ± 14.59	63.6 ± 11.88	.135
Gestational week	38.9 ± 0.66	38.2 ± 1.21	38.9 ± 0.76	.239
Gravidity (n)	2.3 ± 0.92	2.5 ± 1.16	2 ± 0.69	.090
Parity (n)	1.2 ± 0.94	1.3 ± 0.92	1 ± 0.69	.289

Data are presented as mean ± SD and patient numbers. ASA, American Society of Anesthesia; RS, rectus sheath; TAP, transversus abdominis plane.
